# Thermoresponsive C22 phage stiffness modulates the phage infectivity

**DOI:** 10.1038/s41598-022-16795-y

**Published:** 2022-07-29

**Authors:** Udom Sae-Ueng, Anjana Bhunchoth, Namthip Phironrit, Alongkot Treetong, Chaweewan Sapcharoenkun, Orawan Chatchawankanphanich, Ubolsree Leartsakulpanich, Penchit Chitnumsub

**Affiliations:** 1 National Center for Genetic Engineering and Biotechnology (BIOTEC), National Science and Technology Development Agency (NSTDA), Pathum Thani, 12120 Thailand; 2 National Nanotechnology Center (NANOTEC), National Science and Technology Development Agency (NSTDA), Pathum Thani, 12120 Thailand

**Keywords:** Biophysics, Biotechnology, Nanoscience and technology

## Abstract

Bacteriophages offer a sustainable alternative for controlling crop disease. However, the lack of knowledge on phage infection mechanisms makes phage-based biological control varying and ineffective. In this work, we interrogated the temperature dependence of the infection and thermo-responsive behavior of the C22 phage. This soilborne podovirus is capable of lysing *Ralstonia solanacearum*, causing bacterial wilt disease. We revealed that the C22 phage could better infect the pathogenic host cell when incubated at low temperatures (25, 30 °C) than at high temperatures (35, 40 °C). Measurement of the C22 phage stiffness revealed that the phage stiffness at low temperatures was 2–3 times larger than at high temperatures. In addition, the imaging results showed that more C22 phage particles were attached to the cell surface at low temperatures than at high temperatures, associating the phage stiffness and the phage attachment. The result suggests that the structure and stiffness modulation in response to temperature change improve infection, providing mechanistic insight into the C22 phage lytic cycle. Our study signifies the need to understand phage responses to the fluctuating environment for effective phage-based biocontrol implementation.

## Introduction

Bacterial wilt disease caused by *Ralstonia solanacearum* has destroyed economic crops including potato, tomato, tobacco, and chili, costing about 1 billion US dollars per year^1^. Conventional practices such as soil disinfection, soil amendment, and crop rotation are labor-intensive and ineffective due to pathogenic bacteria’s ability to survive in the soil for a long time and propagate to nearby regions via water channels^2,3^. Using chemicals and pesticides to eliminate the bacteria inflicts harmful residues on consumable products, human health, and the environment. Therefore, biological control of *Ralstonia solanacearum* causing wilt disease using antagonistic agents has gained interest as a safe alternative^4,5^. Among such agents, lytic bacteriophages or phages demonstrate promising results in controlling such damaging bacteria^6–9^. Phages target specific host bacterial cells. Once all host cells are eradicated, phages will no longer be replicated. Phages are generally recognized as safe (GRAS) and non-toxic to eukaryotes, rendering them an attractive choice for preventing and treating crop diseases, including bacterial wilt disease^10,11^.

Despite the phage advantages, phage-mediated biocontrol is underutilized due to its variable efficacy^12–15^. The success of phage biocontrol depends on the infection of the bacterial host cell by phage, which is governed by several surrounding factors such as pH, ions, and osmotic pressure^16–18^. An influential factor in all microenvironments is temperature. Phages used in biocontrol constantly experience fluctuating temperatures due to daily climate and seasonal variations in the agricultural fields. The temperature dependence of phages has been previously investigated. However, the exact role of the temperature on phage infection remains unclear. Some phages can infect better at their specific permissive temperatures^19–22^. Lambda phage infecting *Escherichia coli* and the phages infecting *Burkholderia pseudomallei* infected their host cells more effectively and produced more progeny phages at about 35–40 °C than at about 20–35 °C^20,22^. The cell lysis study of *Pseudomonas fluorescens* by ɸS1 phage showed a higher infection rate at 26 °C than 37 °C^23^. On the other hand, some phages are insensitive to temperature. The T4 myovirus phage effectively lysed *E. coli* BL21 within a temperature range of 15 to 41 °C^24^. The P100 phage infecting *Listeria monocytogenes* remained infectious at 4–60 °C^25,26^. The MR5 phage infecting *Pseudomonas syringae,* causing bacterial canker, showed similar infectivity at 20, 27, and 37 °C^27^. Understanding how the temperature affects the phage infection can provide a guideline on phage biocontrol use when coupled with temperature forecast data and infection^6,28^. Therefore, we attempt to decipher the role of the temperature for phages in biocontrol use.

The influence of temperature on phage infection is regulated by how structural components of phages molecularly respond to temperature changes. Infecting *Listeria monocytogenes*, the A511 phage was more stable at high temperatures (60–80 °C) than the P100 phage due to the slightly higher melting point of the A511phage’s tail-capsid connector protein^25^*.* A study on the T7 phage showed that high temperatures reduced the phage infection of the host tail since the high temperature caused the breaking of the phage tail from the phage capsid^29^. Some studies suggested that temperature increase improved infection since it provided more thermal energy to propel the genome into the host cells^30–32^. Understanding the molecular interactions within phage proteins and structures in response to thermal change reveals the underlying mechanism of phage infection and survival. Therefore, insight at the molecular scale will be required to understand the effect of the temperature, which will offer tailor-made guidelines for using phages as a biocontrol agent.

In this work, we revealed the role of temperature on a lytic C22 phage and its infection. The C22 podovirus isolated from a soil sample in Thailand can lyse pathogenic bacteria *Ralstonia solanacearum* (*Rs*), causing bacterial wilt disease in tomato and pepper^33^. The disease also destroys other Solanaceous crops such as potato, tobacco, and eggplant, leading to a global loss of one billion US dollars per year^34^. The C22 phage, therefore, presents a promising and urgently needed antibacterial agent for the bacterial wilt disease. We studied the temperature effect on the C22 phage using a plaque-based infectivity assay. We found that the C22 phage heated at 25–30 °C can infect the host cell better than the C22 phage incubated at 35–40 °C by about 44%. We investigated the direct effect of the temperature by examining the C22 phage particles incubated at different temperatures using atomic force microscopy (AFM)-based techniques. AFM utilizes a cantilever with a nanometer-size tip to caress the phage particles weakly attached on a smooth surface. It does not require any chemical modification or staining on the phage particles, which may hinder some particle details or affect their properties^35–37^. We captured the images of the C22 phages and measured the phage stiffness with AFM-based nano-indentation in a liquid milieu at different temperatures. The results suggest that phage stiffness may play a crucial role in phage binding on the host cell’s surface. Since phage stiffness emerges from the structural integrity of the phage particle, our results signify the thermo-responsive relationship between phage structure and function in the lytic cycle, which must be thoroughly understood for practical phage usage.

## Results

### Temperature dependence of the C22 phage infectivity

We examined the temperature effect on the C22 phage infectivity using a plaque-based infectivity assay (plaque assay) with a slight modification^38^. In our modified plaque assay, the C22 phage was incubated at four temperatures (25, 30, 35, and 40 °C) for 30 min before mixing with the *Rs* cells and incubating on CPG medium agar. After overnight incubation, the number of clear zones or plaques indicating successful lysis of bacterial cells by phage was recorded as plaque forming unit per phage volume (PFU/mL) termed titer. The phage titers at 25, 30, 35, and 40 °C were collected and normalized to 100% by the average phage titer recorded at 25 °C. The temperature range in our study covered a temperature fluctuation, which the C22 phage must endure in the agricultural fields in Thailand^39,40^.

**Figure 1:**
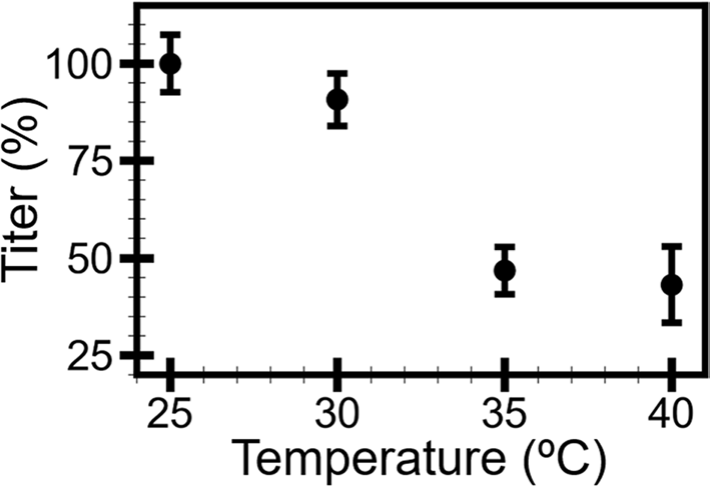
Relative titers of C22 phage at 25, 30, 35, and 40 °C. The titer measurement was conducted in triplicate, and the error bar represented standard deviations (s.d.).

The normalized titers (Fig. 1) exhibited an overall decreasing trend as the temperature increased. When the temperature increased from 25 to 30 °C, the titers slightly decreased by about 10% (see Supplementary Table S1). The titers rapidly decreased by about 44% when the temperature increased from 30 to 35 °C. Finally, as the temperature increased from 35 to 40 °C, the titers remained relatively similar. The result showed that the 30-min incubation caused some alteration to the C22 phage sample and led to the change in infectivity. Therefore, we investigated further how the C22 phage may have been affected during the incubation at each temperature.

### Temperature-induced alteration on C22 phage particles

Some properties of the C22 phage may be thermally changed or induced during the incubation period, causing the titer to decrease. We, therefore, investigated the C22 phage particles at 25, 30, 35, and 40 °C using AFM-based techniques in an MES buffer milieu. The AFM captured a 16-µm^2^ area, which contained more than 10 phage particles. The phage particles were counted and categorized as the following. Individually separated particles were counted into the “dispersed” category. The collections of 2 or more attached particles were counted in the “aggregation” category. The counts in each category were normalized to their total particle counts, which were at least 1000 particles at each temperature (Supplementary Fig. S1). The AFM images of the C22 phage particles at four temperatures showed that most C22 phage particles (> 90%) were dispersed, and only a small portion (< 10%) of the particles aggregated (Supplementary Fig. S2; Table S2).

Due to a lower degree of motion, aggregated phage particles have a lower probability of colliding and attaching to the host cells than dispersed particles. As a result, phage aggregation may lead to decreased infection of the host cells^41,42^. The analysis of the AFM images showed that the majority of the phage particles dispersed. This result implies that most C22 phage particles incubated at each temperature were available to bind and interact with the host cells. The dispersion and aggregation of the C22 phage at four temperatures were quantitatively similar. Therefore, aggregation was unlikely to cause decreased infectivity observed by the plaque assay. The reduced infection may be instead caused by the change in the properties of individual phage particles.

**Figure 2:**
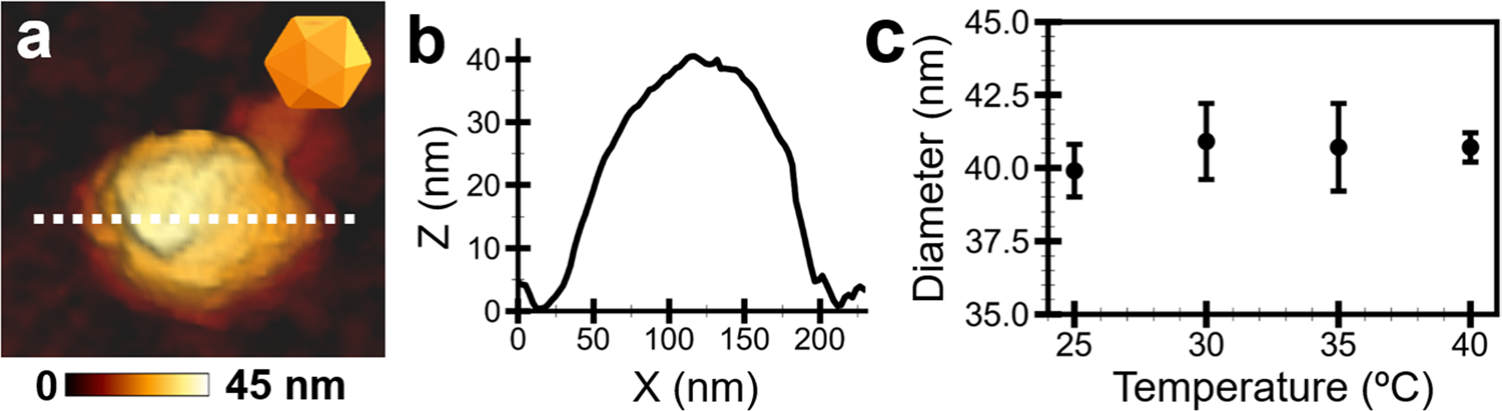
The size of a C22 phage particle examined by AFM. (**a**) AFM image of a C22 phage particle in the MES buffer at 25 °C. The color gradient scale bar indicated the height (Z-direction). The orange inset demonstrated an icosahedral shape. (**b**) The particle diameter was extracted from the particle’s cross-section (white dashed line). (**c**) The average diameter of the C22 phage particles at 25, 30, 35, and 40 °C was about 40 nm. The error bars represented standard deviations (s.d.) of 50 particles.

The structure of the C22 phage particles in the MES buffer at 25, 30, 35, and 40 °C was captured and analyzed by AFM non-contact imaging method. The AFM data and analysis demonstrated that the C22 phage particle remained intact and morphologically similar in the four temperatures. An exemplary AFM image of the C22 phage in MES buffer at 25 °C (Fig. 2a) showed an icosahedral shape with a diameter of about 40 nm (Fig. 2b). The angular characteristic observed for small phage particles became noticeable for the C22 phage^43,44^. No structural damage or disintegration of the phage particles was observed upon a close look at the phage structure at four temperatures (Supplementary Fig. S3). The average diameter of the C22 phage particles was approximately the same at four temperatures (Fig. 2c; Supplementary Table S3), suggesting no shrinking or swelling of the C22 phage particles. Therefore, structural damage or instability of the phage particle is not a cause of the infectivity decrease.

### Stiffness of C22 phage particles at different temperatures

**Figure 3:**
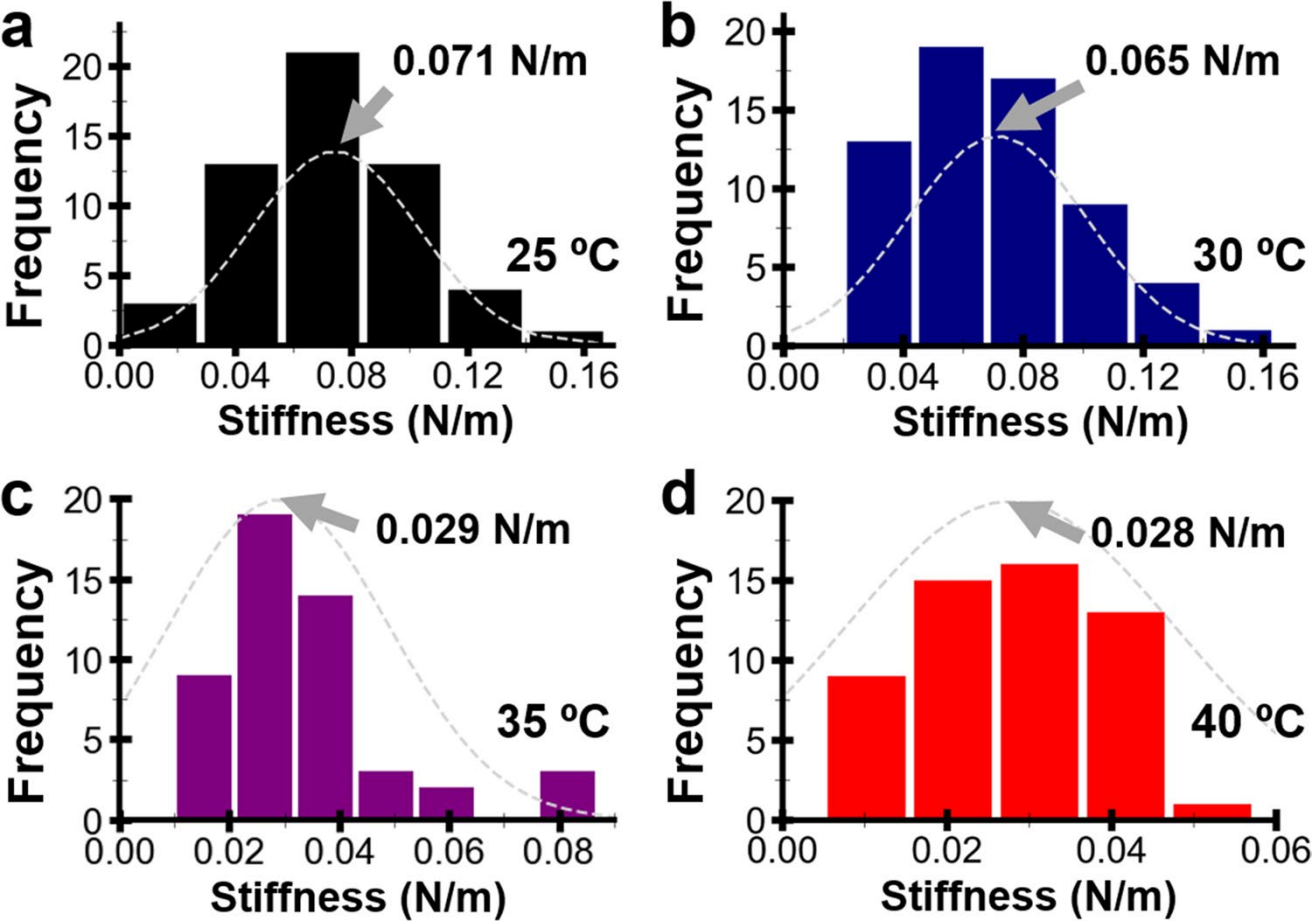
Plots of the stiffness distributions of the C22 phage in MES buffer at 25 (**a**), 30 (**b**), 35 (**c**), and 40 °C (**d**). The dashed grey lines were the normal distribution fitting where the centers (grey arrows) represented the phage stiffness at each temperature.

**Figure 4:**
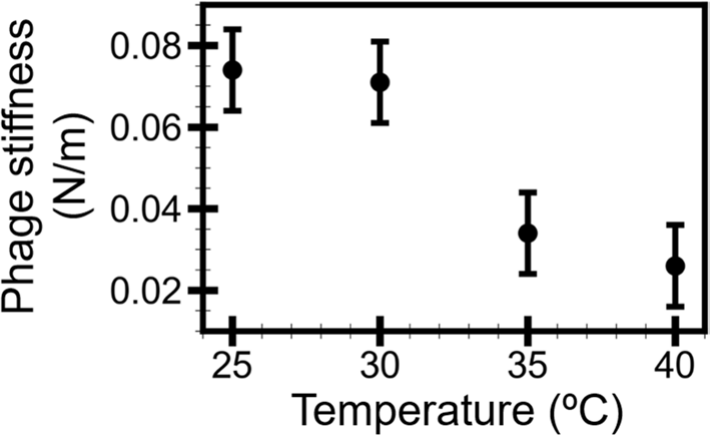
The C22 phage stiffness decreased as the temperature increased from 25 to 40 °C. The error bars represented standard deviation (s.d.).

Although the C22 phage particles were morphologically similar, other structural properties of the phage particles may still alter within 25–40 °C. So, we employed another AFM-based approach termed nano-indentation to probe the dynamical changes of the phage particles. The approach indented the particle and measured the phage’s mechanical properties such as stiffness and breaking force. Such properties are associated with critical steps in the viral life cycle, such as genome packaging^45,46^, genome transfer^47,48^, and capsid stability^29,49^. In this study, the stiffness of the C22 phage particle in MES buffer at 25, 30, 35, and 40 °C was extracted and calculated from force-distance curves of the phage particle and the mica (Supplementary Fig. S4). The distributions of the phage stiffness at 25, 30, 35, and 40 °C were generated from 50 to 60 phage stiffness values at each temperature. The distributions were fitted by normal distribution to extract the representative stiffness of the C22 phage (Fig. 3). The stiffness of the C22 phage showed a decreasing trend as the temperature increased (Fig. 4). The phage stiffness at 30 °C (0.065 ± 0.01 N/m) was slightly lower than at 25 °C (0.071 ± 0.01 N/m). The phage stiffness decreased by about 55% at 35 °C (0.029 ± 0.01 N/m). The phage stiffness remained relatively unaffected at 40 °C (0.028 ± 0.01 N/m). We hypothesized that the stiffness decrease might be associated with the decreased infection of the C22 phage that was observed by the modified plaque assay.

### Binding of C22 phage particles on host cells at different temperatures

An obvious question remains: How does the phage stiffness decrease contribute to the decreased infectivity? To answer this question, we investigated how the C22 phage at different temperatures interacted with the host cell. After being incubated at each temperature for 30 min, the C22 phage was mixed with the host cells with the same multiplicity of infection as the plaque infectivity study. The mixture was investigated with AFM imaging.

**Figure 5:**
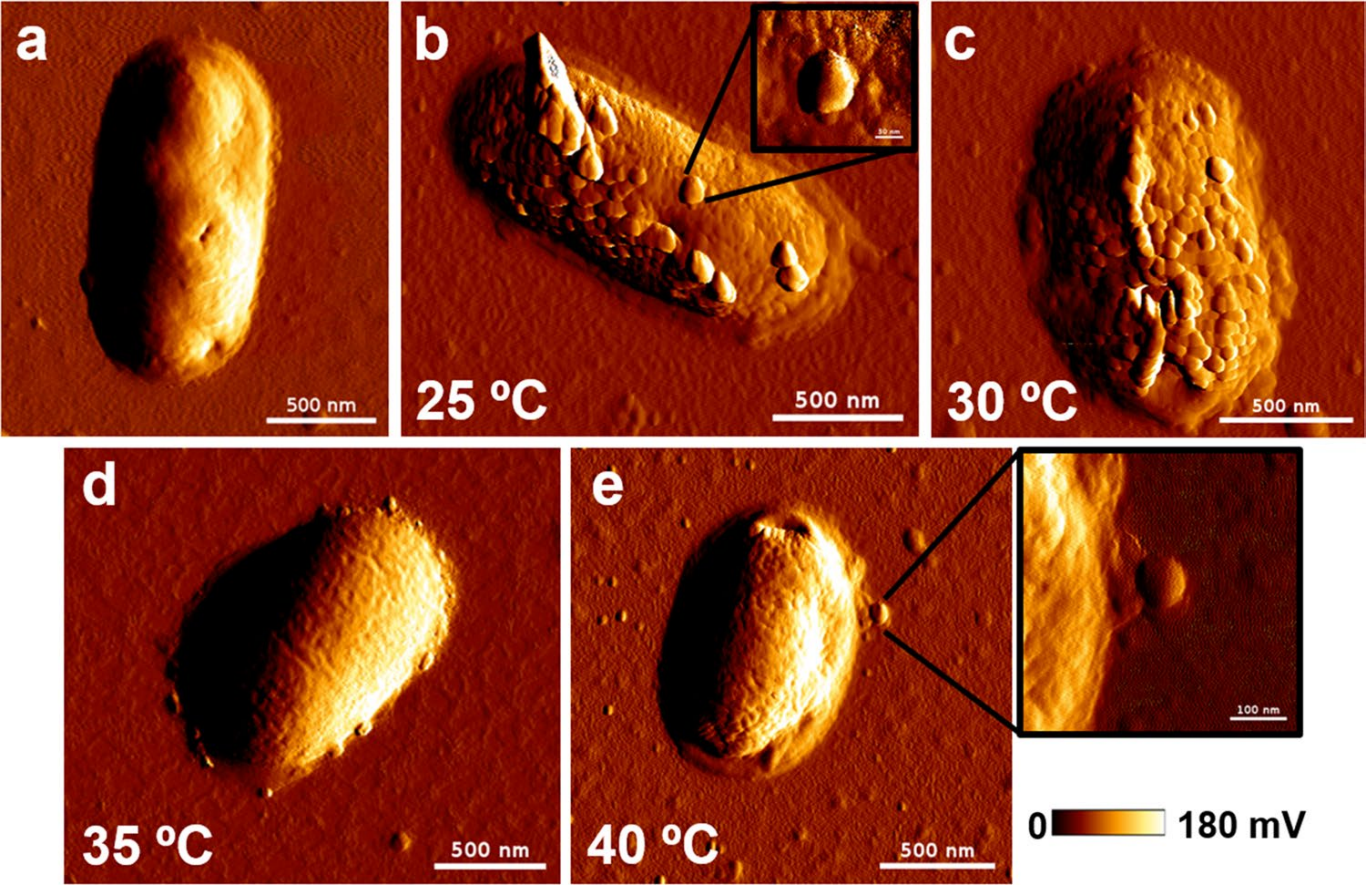
Exemplary AFM images of the C22 phage and *Rs* cells at different temperatures. (**a**) An AFM image of an *Rs* cell without the C22 phage. The C22 phage incubated at 25 °C (**b**), 30 °C (**c**), 35 °C (**d**), and 40 °C (**e**) mixed with *Rs* cells was investigated by AFM. Individual C22 phage particles could be distinguished in (**b**,**e**) insets. The color gradient scale bar was the scale of the vertical amplitude (Z-direction). The white scale bars in the (**a**–**e**) panels were 500 nm, and those in the inset were 100 nm.

The size of an *Rs* cell (Fig. 5a) was measured to be about 1.8 µm in length and 0.9 µm in width. Studying the mixture of the C22 phage and the cells, we found that the phage particles bound on the cell surface could be distinguishably observed (Fig. 5b,e insets). The binding of the C22 phage on the cell surface suggests that the potential receptors are located on the cell surface. The candidate receptors are transmembrane proteins or molecules extruding from the cell surface (e.g., lipopolysaccharide)^31,50^. We also found the different numbers of the C22 phage particles attached to the cell surface. At 25 and 30 °C, many C22 phage particles were bound on the cell surface (Fig. 5b,c), but at 35 and 40 °C, only a few C22 phage particles were bound on the cell surface (Fig. 5d,e). More phage particles attached to the host cell imply a higher chance of the cell being infected by the phage. The data indicates that the C22 phage incubated at 25 and 30 °C could infect the host cell more effectively than at 35 and 40 °C. The data is also concordant with the plaque assay result, which showed an infection improvement at 25 and 30 °C. Combining with the phage stiffness result, we propose that the relatively rigid C22 phage particles at 25 and 30 °C could bind to the cell surface and infect the host cell better than the relatively soft C22 phage particles at 35 and 40 °C.

## Discussion

In this work, we found that the infection of the *Rs* cells by the C22 phage decreased as we incubated the C22 phage at increasing temperatures from 25 to 40 °C. The temperature must cause some changes to the C22 phage particles within this temperature range, resulting in decreasing infectivity. Upon inspection, the phage particles were primarily non-aggregated, eliminating aggregation as a likely cause of reduced infection. Further investigation of individual phage particles revealed that the particles did not show morphological changes when incubated at increasing temperatures; however, the stiffness of the particles was reduced. Increasing thermal energy could enhance the local vibration of capsid proteins and DNA, weakening attractive interaction among capsomeres and double-stranded (ds) DNA strands, thus decreasing stiffness^51^. Previous computational studies suggested that viral capsids may undergo a “fluidity” transition with increasing temperature^52^. Under this transition, the average separation between capsomeres goes over a specific threshold, expressing more capsid flexibility and lower stiffness. A decrease in densely-packed dsDNA stiffness can also play a part in reduced phage stiffness due to a solid-to-fluid-like transition of dsDNA inside viral capsids^32,48^. The DNA structure became more disordered and showed decreased stiffness when the temperature increased. Altogether, the C22 phage capsid and the internal dsDNA probably experience similar transitions, contributing to the decreased stiffness.

We speculated that the decreasing stiffness regulated the infection of the *Rs* cell. The discovery that the decreasing stiffness was associated with the reduced number of phage particles attached to the cell membrane strengthened our hypothesis. In other words, the thermally induced decreasing stiffness of the C22 phage particle hinders the binding efficacy, thus reducing the infection of the host cell by the C22 phage. The finding urges us to explore the phage stiffness’s role in the phage binding in the infection cycle. A classical model of phage adsorption depicts that the binding is initiated by the recognition between the phage tail fibers or spikes and the host cell receptor^53,54^. The phage stiffness primarily emanates from the capsid protein and internally packed dsDNA due to their abundance^32,45,55,56^. Therefore, the phage stiffness’s effect may originate from the capsid proteins and DNA and propagate to the fiber and spike proteins. We hypothesize that portal proteins may perform this proposed mechanistic dissemination^57,58^. This dynamical structure connecting the capsid-DNA ensemble and the tail proteins has been shown to be flexible and behave like a mechanical sensor that detects the overall structural alteration^59,60^. A higher stiffness phage particle infers a more compact capsid structure and tightly packed DNA^61^. Such a structure may possess a smaller intermolecular distance and, in turn, mechanically propagate the structural changes better than a lower stiffness phage particle. For example, the movement of tail fibers or spikes during binding can be promoted. The DNA ejection can be driven by the propagation of portal protein extension or compression better in a higher stiffness than lower stiffness phage particle^62,63^. The precise mechanisms at the molecular level require further structural study, which will be pursued in future work.

Furthermore, the temperature may influence phage infectivity through additional mechanisms. The temperature can induce conformational changes or may damage fiber and spike proteins affecting their properties (e.g., flexibility) and the virus binding^19,64^. The temperature can also increase the phage particles’ stochastic motion, influencing the binding and releasing events between the phage binding proteins and the host cell receptors^65,66^. The temperature can affect the expression of genes or proteins involved in the infection process^67,68^. However, the extent of the temperature effect on structural components is unclear due to the transient nature of the phage-host interaction.

In our recent study, the stiffness of the C22 phage varied with different pH and ionic strengths, but the adsorption efficiency remained relatively constant^35^. The AFM results in this study revealed otherwise. Upon increasing temperature, the number of the C22 phage particles in the adsorption step was reduced, which can be linked to the decreasing stiffness of the C22 phage particle. This discrepancy is likely due to the methods used for investigating phage adsorption. The previous study relied on the quantity of the unadsorbed phage particles in the supernatant after precipitation of the phage-adsorbed cells. This experimental approach did not directly probe the adsorption of the phage particles on the cell surface. In this study, we directly visualized the C22 phage binding on the host cell, which provides the actual view of the in situ binding events with higher resolution and accuracy.

The dynamics of viral stiffness result from molecular adjustment of the virus particle to maximize its chance of infection. The stiffness modulation likely depends on the virus type and, in turn, the responsive structural components. Our previous study showed that the high infectivity of the C22 phage is associated with its intermediate stiffness^35^. The minute virus of mice relaxed the capsomeres on the capsid and capsomere-tail portal linkage, leading to lower mechanical stiffening and improved infection^69^. Lambda phage and herpes simplex virus type 1 promoted DNA transfer into host cells by decreasing DNA stiffness^32,48,70,71^. The human immunodeficiency virus reduced its membrane stiffness via maturation to improve cell entry^72^. Therefore, stiffness regulation can be considered a feedback mechanism for viruses to adjust for survival and replication.

Our results signify the thermo-responsive structural and nanomechanical aspects of the phage lytic cycle, which must be thoroughly understood for practical phage usage in crop disease management. Understanding the C22 phage infection will allow us to devise how the C22 phage should be effectively used in the fields with fluctuating temperatures in biocontrol applications. For example, farmers should use the C22 phage during times with lower temperatures (25–30 °C) since the infectivity of the C22 phage is likely reduced when the temperature reaches 35–40 °C. The knowledge in this study and the availability of precision farming technology such as soil temperature sensors and IoT will help the farmers to monitor the environment status and adequately administer the phage biocontrol^28,73^.

## Methods

### Cell and bacteriophage preparation

An overnight culture of *Ralstonia solanacearum* strain RS3/1-1 (race 1 biovar 4) at 28 °C and 230 rpm shaking was diluted to OD_600_ of 0.25 with fresh CPG medium containing 0.1% (w/v) casamino acids, 1% (w/v) peptone, and 0.5% (w/v) glucose. Purification of the C22 phage was described elsewhere^35^. The C22 phage pellet was resuspended in the MES buffer consisting of 0.05 M 2-(N-Morpholino)ethanesulfonic acid or MES (Sigma-Aldrich) pH 6.0, 0.1 M NaCl, and 0.05 M MgSO_4_ and stored at 4°C^35,74^.

### Plaque-based infectivity assay

An overnight culture of *Rs* strain RS3/1-1 at 28 °C and 230 rpm shaking was diluted to OD_600_ of 0.25 with fresh CPG medium. For the first case (incubated phage), 100 μL of the C22 phage in MES buffer was incubated at each temperature (25, 30, 35, 40 °C) for 30 min. After that, 10 μL of the incubated phage suspension was mixed with 250 μL of diluted *Rs* culture. After a 30-min incubation, the mixture was added with molten 0.45% agar in a CPG medium and overlaid on a CPG plate containing 1.5% agar. After overnight incubation at 28 °C, the number of plaques was recorded. The plaque assay was performed in triplicate.

### Substrate preparation for atomic force microscope

Mica (V1 grade, Ted Pella) was separately coated with two chemicals. The first chemical was a poly-l-lysine (PLL) solution (Sigma-Aldrich). A mica was cleaved and incubated in a 0.1% (w/v) PLL solution for 15 min. The mica was rinsed with deionized water and dried with nitrogen gas. A PLL-coated mica was used to immobilize *Rs* cells. The second chemical was (3-Aminopropyl) triethoxysilane (APTS) (Sigma-Aldrich). A mica was cleaved and incubated in a 1% APTS solution in deionized water with 50 rpm shaking for 15 min. The mica was rinsed with deionized water and dried with nitrogen gas. An APTS-coated mica was used to immobilize the C22 phage particles.

### AFM-based imaging and nano-indentation

For the *Rs* cell AFM imaging, the C22 phage was incubated at each temperature (25, 30, 35, 40 °C) for 30 min. The incubated phage was then mixed with the *Rs* cell for 30 min and subsequently incubated on the PLL-coated mica for 30 min at room temperature. After that, the immobilized cells on the mica substrate were washed with deionized water and dried with nitrogen gas. AFM imaging measurements were performed with a NanoWizard 3 BioScience AFM (JPK-Bruker). The images were collected in the non-contact mode (AC mode) under ambient conditions. During the imaging, the AFM cantilever (ACTA model, AppNano) with a nanometer-size tip at the end oscillated as the cantilever scanned on the sample surface (the X and Y axes). The responsive oscillation amplitude in the vertical (Z) axis was recorded as the amplitude images of the sample surface.

For the C22 phage AFM imaging and nano-indentation, a 100 μL droplet of the C22 phage in MES buffer with (~ 10^12^ pfu/mL) was incubated at each temperature (25, 30, 35, 40 °C) for 30 min. A 50 μL droplet of the incubated phage suspension was incubated with the APTS-treated mica substrate for 30 min before the AFM experiment. The temperature of the AFM sample checked by a digital thermometer (421TK, Ponpe Instruments) was kept constant at each temperature with a heating stage (PetriDish Heater, JPK-Bruker). Images of the C22 phage were captured in the non-contact mode (AC mode). The stiffness of the phage particles was measured under the force spectroscopy mode of the AFM. A BioLever mini BL-AC40TS-C2 (Olympus) cantilever was used with the spring constant of 0.02–0.14 N/m determined by thermal tuning. Briefly, the force-distance (FD) curve recorded the indentation of the AFM tip into the center region of the phage particle. The slopes extracted from the linear region of the FD curves on the particle and the mica were calculated to the spring constant or stiffness of the phage particle (Supplementary Fig. S3). The calculation details were described elsewhere^32,45,45,75^.

### AFM data analysis

At each temperature (25, 30, 35, 40 °C), 50–60 stiffness values were measured from 15 to 20 phage particles to generate a stiffness distribution. A Gaussian function (normal distribution) was fitted to the empirical stiffness distribution using the normal probability density function, open-source software in Python^76^. The peak center representing the phage stiffness and the standard deviation (s.d.) were extracted from the fitting. The standard deviation was then calculated to the standard error (s.e.m.) of the stiffness^75,77^. All of the plot figures in the manuscript were generated by the Mathplotlib library in Python^78^.

## Supplementary Information

Supplementary Information.

## Data Availability

All data generated or analyzed during this study are included in this published article and its supplementary information file.

## References

[1] *Bacterial Wilt Disease and the Ralstonia Solanacearum Species Complex. Bacterial Wilt Disease and the Ralstonia Solanacearum Species Complex*. (American Phytopathological Society (APS Press), 2005).

[2] Yuliar X, Nion YA, Toyota K (2015). Recent trends in control methods for bacterial wilt diseases caused by *Ralstonia solanacearum*. Microbes Environ..

[3] Saddler GS, Allen C, Prior P, Hayward AC (2005). Management of bacterial wilt disease. Bacterial Wilt Disease and the Ralstonia solanacearum Species Complex.

[4] Elsayed TR, Jacquiod S, Nour EH, Sørensen SJ, Smalla K (2020). Biocontrol of bacterial wilt disease through complex interaction between tomato plant, antagonists, the indigenous rhizosphere microbiota, and *Ralstonia solanacearum*. Front. Microbiol..

[5] Saputra R, Arwiyanto T, Wibowo A (2020). Biological control of *Ralstonia solanacearum* causes of bacterial wilt disease with *Pseudomonas putida* and *Streptomyces* spp. on some tomato varieties. IOP Conf. Ser. Earth Environ. Sci..

[6] Holtappels D, Fortuna K, Lavigne R, Wagemans J (2021). The future of phage biocontrol in integrated plant protection for sustainable crop production. Curr. Opin. Biotechnol..

[7] Álvarez B, Biosca EG (2017). Bacteriophage-based bacterial wilt biocontrol for an environmentally sustainable agriculture. Front. Plant Sci..

[8] Dy RL, Rigano LA, Fineran PC (2018). Phage-based biocontrol strategies and their application in agriculture and aquaculture. Biochem. Soc. Trans..

[9] Fujiwara A (2011). Biocontrol of *Ralstonia solanacearum* by treatment with lytic bacteriophages. Appl. Environ. Microbiol..

[10] Ul Haq I, Chaudhry WN, Akhtar MN, Andleeb S, Qadri I (2012). Bacteriophages and their implications on future biotechnology: A review. Virol. J..

[11] Kazi M, Annapure US (2016). Bacteriophage biocontrol of foodborne pathogens. J. Food Sci. Technol..

[12] Hassan AY, Lin JT, Ricker N, Anany H (2021). The age of phage: Friend or foe in the new dawn of therapeutic and biocontrol applications?. Pharmaceuticals.

[13] Cristobal-Cueto P, García-Quintanilla A, Esteban J, García-Quintanilla M (2021). Phages in food industry biocontrol and bioremediation. Antibiotics.

[14] Wang X (2019). Phage combination therapies for bacterial wilt disease in tomato. Nat. Biotechnol..

[15] Buttimer C (2017). Bacteriophages and bacterial plant diseases. Front. Microbiol..

[16] Ramirez K, Cazarez-Montoya C, Lopez-Moreno HS, Castro-del Campo N (2018). Bacteriophage cocktail for biocontrol of *Escherichia coli* O157:H7: Stability and potential allergenicity study. PLoS One.

[17] Ly-Chatain MH (2014). The factors affecting effectiveness of treatment in phages therapy. Front. Microbiol..

[18] Jończyk E, Kłak M, Międzybrodzki R, Górski A (2011). The influence of external factors on bacteriophages-review. Folia Microbiol. (Praha).

[19] Gale P (2020). How virus size and attachment parameters affect the temperature sensitivity of virus binding to host cells: Predictions of a thermodynamic model for arboviruses and HIV. Microb. Risk Anal..

[20] Egilmez HI (2018). Temperature-dependent virus lifecycle choices may reveal and predict facets of the biology of opportunistic pathogenic bacteria. Sci. Rep..

[21] Shan J (2014). Temperature dependent bacteriophages of a tropical bacterial pathogen. Front. Microbiol..

[22] Moldovan R, Chapman-McQuiston E, Wu XL (2007). On kinetics of phage adsorption. Biophys. J..

[23] Sillankorva S, Oliveira R, Vieira MJ, Sutherland I, Azeredo J (2004). Pseudomonas fluorescens infection by bacteriophage ΦS1: the influence of temperature, host growth phase and media. FEMS Microbiol. Lett..

[24] Taj M (2014). Effect of dilution, temperature and pH on the lysis activity of T4 phage against *E. coli* BL21. J. Anim. Plant Sci..

[25] Ahmadi H, Radford D, Kropinski AM, Lim L-T, Balamurugan S (2017). Thermal-stability and reconstitution ability of listeria phages P100 and A511. Front. Microbiol..

[26] Fister S (2016). Influence of environmental factors on phage-bacteria interaction and on the efficacy and infectivity of phage P100. Front. Microbiol..

[27] Rabiey M (2020). Phage biocontrol to combat *Pseudomonas syringae* pathogens causing disease in cherry. Microb. Biotechnol..

[28] Mamphogoro TP, Babalola OO, Aiyegoro OA (2020). Sustainable management strategies for bacterial wilt of sweet peppers (*Capsicum annuum*) and other Solanaceous crops. J. Appl. Microbiol..

[29] Vörös Z, Csík G, Herényi L, Kellermayer M (2018). Temperature-dependent nanomechanics and topography of bacteriophage T7. J. Virol..

[30] Zhang C-Y, Zhang N-H (2020). Size effect on structure and stiffness of viral DNA during temperature variation. bioRxiv.

[31] Broeker NK (2018). In vitro studies of lipopolysaccharide-mediated DNA release of podovirus HK620. Viruses.

[32] Liu T (2014). Solid-to-fluid-like DNA transition in viruses facilitates infection. Proc. Natl. Acad. Sci. USA.

[33] Bhunchoth A (2015). Isolation of *Ralstonia solanacearum*-infecting bacteriophages from tomato fields in Chiang Mai, Thailand, and their experimental use as biocontrol agents. J. Appl. Microbiol..

[34] Elphinstone JG (2005). The current bacterial wilt situation: A global overview. Bacterial wilt Disease and the Ralstonia solanacearum Species Complex.

[35] Sae-Ueng U (2020). C22 podovirus infectivity is associated with intermediate stiffness. Sci. Rep..

[36] Dufrêne YF (2017). Imaging modes of atomic force microscopy for application in molecular and cell biology. Nat. Nanotechnol..

[37] Alsteens D, Trabelsi H, Soumillion P, Dufrêne YF (2013). Multiparametric atomic force microscopy imaging of single bacteriophages extruding from living bacteria. Nat. Commun..

[38] Kropinski AM, Mazzocco A, Waddell TE, Lingohr E, Johnson RP (2009). Enumeration of bacteriophages by double agar overlay plaque assay. Methods Mol. Biol..

[39] Tuntiwaranuruk U, Sriboon W (2018). The influence of ambient temperature on soil temperature in concrete containers with lime trees. NU. Int. J. Sci..

[40] Sriboon W, Tuntiwaranuruk U, Sanoamuang N (2017). Hourly soil temperature and moisture content variations within a concrete pipe container for planting lime trees in Eastern Thailand. Case Stud. Therm. Eng..

[41] Pradhan S, Varsani A, Leff C, Swanson CJ, Hariadi RF (2022). Viral aggregation: The knowns and unknowns. Viruses.

[42] Szermer-Olearnik B (2017). Aggregation/dispersion transitions of T4 phage triggered by environmental ion availability. J. Nanobiotechnol..

[43] Sanz-Gaitero M, Seoane-Blanco M, van Raaij MJ (2019). Structure and function of bacteriophages. Bacteriophages.

[44] Zandi R, Reguera D, Bruinsma RF, Gelbart WM, Rudnick J (2004). Origin of icosahedral symmetry in viruses. Proc. Natl. Acad. Sci. USA.

[45] Ivanovska I, Wuite G, Jönsson B, Evilevitch A (2007). Internal DNA pressure modifies stability of WT phage. Proc. Natl. Acad. Sci. USA.

[46] Sae-Ueng U (2014). Major capsid reinforcement by a minor protein in herpesviruses and phage. Nucleic Acids Res..

[47] Kellermayer MSZ, Vörös Z, Csík G, Herényi L (2018). Forced phage uncorking: Viral DNA ejection triggered by a mechanically sensitive switch. Nanoscale.

[48] Sae-Ueng U (2014). Solid-to-fluid DNA transition inside HSV-1 capsid close to the temperature of infection. Nat. Chem. Biol..

[49] Llauró A, Schwarz B, Koliyatt R, De Pablo PJ, Douglas T (2016). Tuning viral capsid nanoparticle stability with symmetrical morphogenesis. ACS Nano.

[50] Chaturongakul S, Ounjai P (2014). Phage-host interplay: examples from tailed phages and Gram-negative bacterial pathogens. Front. Microbiol..

[51] van de Waterbeemd M (2017). Structural analysis of a temperature-induced transition in a viral capsid probed by HDX-MS. Biophys. J..

[52] Singh AR, Košmrlj A, Bruinsma R (2020). Finite temperature phase behavior of viral capsids as oriented particle shells. Phys. Rev. Lett..

[53] Nobrega FL (2018). Targeting mechanisms of tailed bacteriophages. Nat. Rev. Microbiol..

[54] Fokine A, Rossmann MG (2014). Molecular architecture of tailed double-stranded DNA phages. Bacteriophage.

[55] de Pablo PJ (2018). Atomic force microscopy of virus shells. Semin. Cell Dev. Biol..

[56] Roos WH, Bruinsma R, Wuite GJL (2010). Physical virology. Nat. Phys..

[57] Rao VB, Fokine A, Fang Q (2021). The remarkable viral portal vertex: structure and a plausible model for mechanism. Curr. Opin. Virol..

[58] Prevelige PE, Cortines JR (2018). Phage assembly and the special role of the portal protein. Curr. Opin. Virol..

[59] Dedeo CL, Cingolani G, Teschke CM (2019). Portal protein: The orchestrator of capsid assembly for the dsDNA tailed bacteriophages and herpesviruses. Annu. Rev. Virol..

[60] Kumar R, Grubmüller H (2014). Elastic properties and heterogeneous stiffness of the Phi29 motor connector channel. Biophys. J..

[61] Carrasco C, Castellanos M, De Pablo PJ, Mateu MG (2008). Manipulation of the mechanical properties of a virus by protein engineering. Proc. Natl. Acad. Sci. USA.

[62] Chaban Y (2015). Structural rearrangements in the phage head-to-tail interface during assembly and infection. Proc. Natl. Acad. Sci. USA.

[63] Zairi M, Stiege AC, Nhiri N, Jacquet E, Tavares P (2014). The collagen-like protein gp12 is a temperature-dependent reversible binder of SPP1 viral capsids. J. Biol. Chem..

[64] Shi J, Shen Q, Cho JH, Hwang W (2020). Entropy hotspots for the binding of intrinsically disordered ligands to a receptor domain. Biophys. J..

[65] Baptista C, Santos MA, São-José C (2008). Phage SPP1 reversible adsorption to Bacillus subtilis cell wall teichoic acids accelerates virus recognition of membrane receptor YueB. J. Bacteriol..

[66] Baxa U (1996). Interactions of phage P22 tails with their cellular receptor, Salmonella O-antigen polysaccharide. Biophys. J..

[67] Bisht K, Moore JL, Caprioli RM, Skaar EP, Wakeman CA (2021). Impact of temperature-dependent phage expression on *Pseudomonas aeruginosa* biofilm formation. NPJ Biofilms Microbiomes.

[68] Ghosh S, Shaw R, Sarkar A, Gupta SKD (2020). Evidence of positive regulation of mycobacteriophage D29 early gene expression obtained from an investigation using a temperature-sensitive mutant of the phage. FEMS Microbiol. Lett..

[69] Guerra P (2017). Structural basis for biologically relevant mechanical stiffening of a virus capsid by cavity-creating or spacefilling mutations. Sci. Rep..

[70] Brandariz-Nuñez A, Liu T, Du T, Evilevitch A (2019). Pressure-driven release of viral genome into a host nucleus is a mechanism leading to herpes infection. Elife.

[71] Bauer DW, Evilevitch A (2015). Influence of internal DNA pressure on stability and infectivity of phage λ. J. Mol. Biol..

[72] Kol N (2007). A stiffness switch in human immunodeficiency virus. Biophys. J..

[73] Álvarez B, López MM, Biosca EG (2019). Biocontrol of the major plant pathogen *Ralstonia solanacearum* in irrigation water and host plants by novel waterborne lytic bacteriophages. Front. Microbiol..

[74] Manbua N, Suteewong T, Sae-Ueng U (2022). Efficacy of sugar excipients on lyophilized C22 phage infectivity evaluated by atomic force microscopy. Biol. Control.

[75] Ivanovska IL (2004). Bacteriophage capsids: Tough nanoshells with complex elastic properties. Proc. Natl. Acad. Sci. USA.

[76] scipy.stats.norm—SciPy v1.6.2 Reference Guide. https://docs.scipy.org/doc/scipy/reference/generated/scipy.stats.norm.html. .

[77] de Pablo PJ, Mateu MG (2013). Mechanical properties of viruses. Subcell. Biochem..

[78] Matplotlib: Python plotting—Matplotlib 3.4.1 documentation. https://matplotlib.org/.

