# Supplementary material for: Thermoresponsive C22 phage stiffness modulates the phage infectivity
Source: Sci Rep. 2022 Jul 29;12:13001. doi: 10.1038/s41598-022-16795-y (PMC9338302; doi:10.1038/s41598-022-16795-y)

Supplementary data

**Thermoresponsive C22 phage stiffness modulates the phage infectivity**

Udom Sae-Ueng^1*^, Anjana Bhunchoth^1^, Namthip Phironrit^1^, Alongkot Treetong^2^,

Chaweewan Sapcharoenkun^2^, Orawan Chatchawankanphanich^1^, Ubolsree Leartsakulpanich^1^, and Penchit Chitnumsub^1^

^1^ National Center for Genetic Engineering and Biotechnology (BIOTEC), National Science and Technology Development Agency (NSTDA), Pathum Thani, 12120, Thailand

^2^ National Nanotechnology Center (NANOTEC), National Science and Technology Development Agency (NSTDA), Pathum Thani, 12120, Thailand

^*^ Corresponding author: udom.sae@biotec.or.th

**Supplementary Table S1.** The titers of the C22 phage at 25, 30, 35, and 40 ºC. The titers were normalized by the titer of the C22 phage at 25 ºC. The titer measurement was conducted in triplicate, and the error represented standard deviations (s.d.).

| Temperature (ºC) | Normalized phage titers (%) |
| --- | --- |
| 25 | 100.00 ± 7.38 |
| 30 | 90.71 ± 6.80 |
| 35 | 46.79 ± 6.04 |
| 40 | 43.15 ± 9.77 |

**Supplementary Figure S1.** Dispersion and aggregation of the C22 phage particles observed by AFM (a) An exemplary AFM image of the C22 phage particles at 25 ºC in the MES buffer. The dispersed phage particles were denoted by “d,” and the aggregated phage particles were denoted by “a.” The white scale bar was 1 µm. The color gradient scale bar indicated the height (Z-direction). (b) Relative distributions of dispersed and aggregated C22 phage particles at each temperature. The error bars represented the counting error from the number of phage particles in the AFM images.


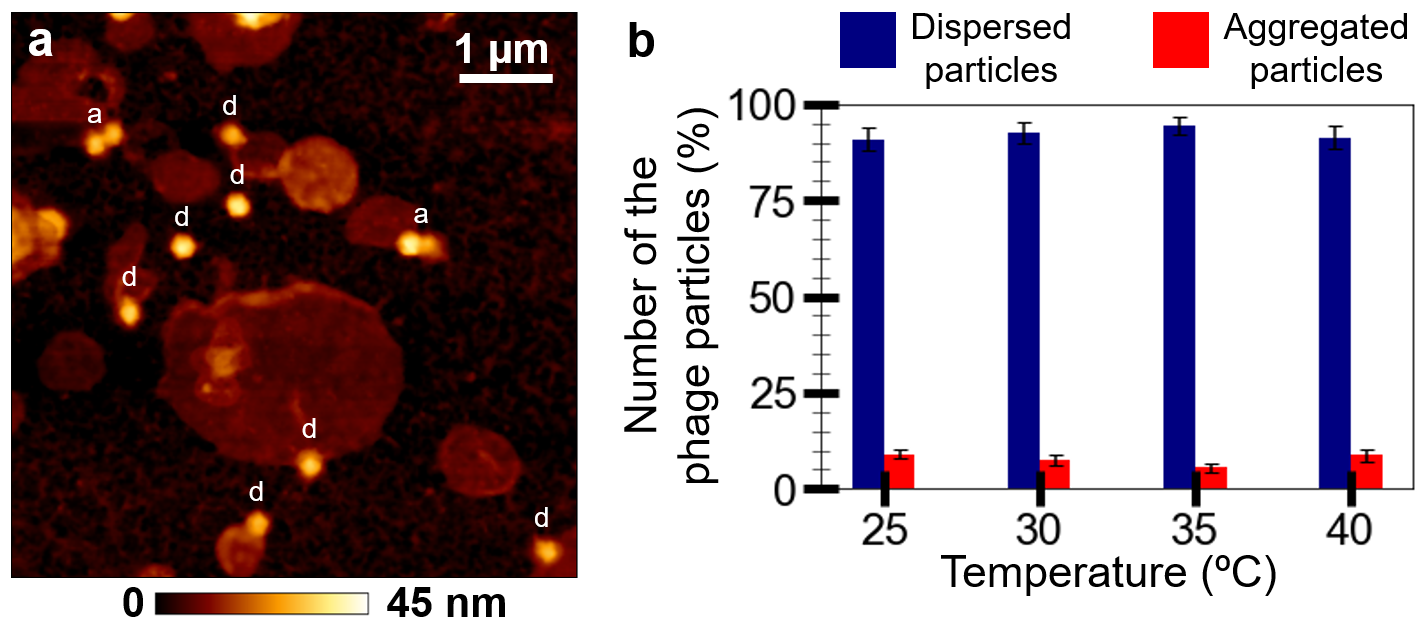


**Supplementary Figure S2.** Exemplary AFM images of the C22 phage in MES buffer at 25 (a), 30 (b), 35 (c), and 40(d) ºC. The color gradient scale bar represented the height (Z-direction scale).


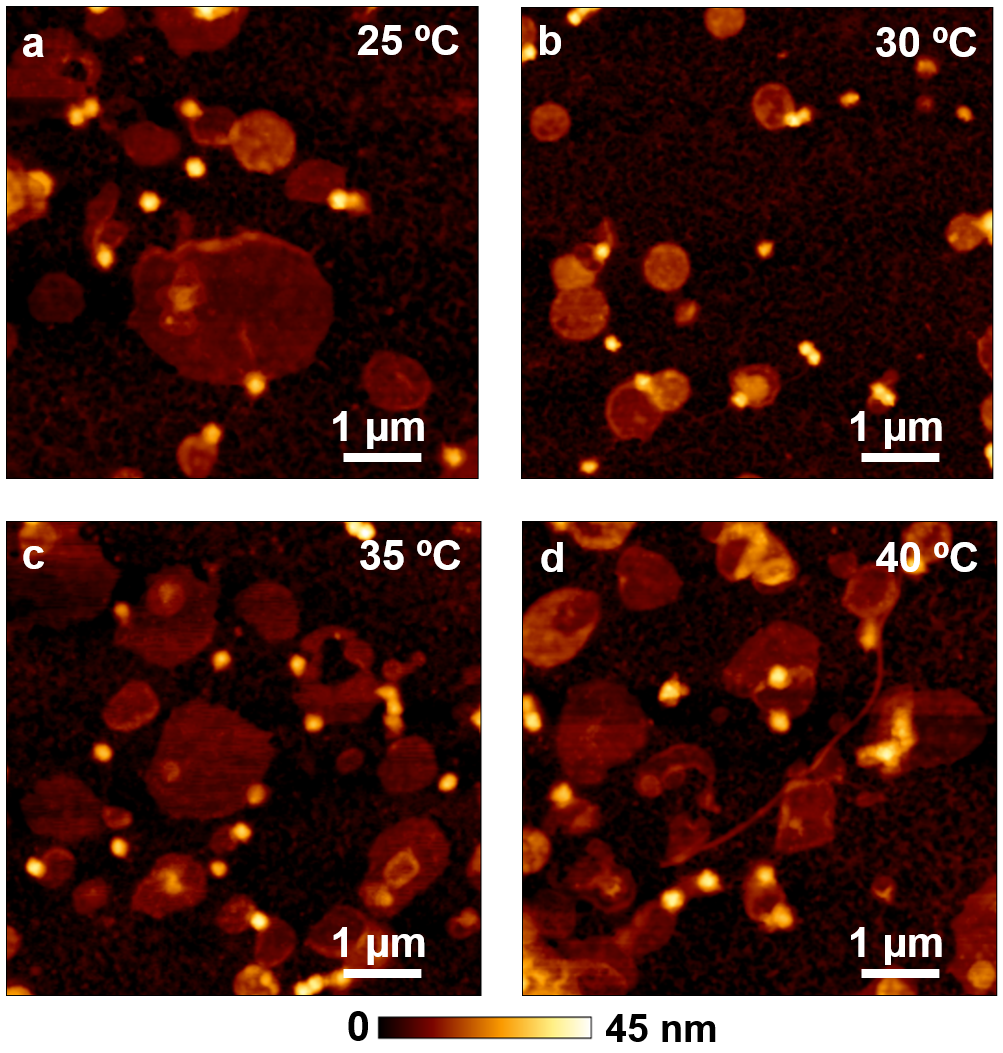


**Supplementary Table S2.** Relative portions of dispersed and aggregated C22 phage particles in the MES buffer at 25, 30, 35, and 40 ºC. The counts in each category were normalized to their total particle counts at each temperature. The error represented the counting error from the number of phage particles in the AFM images.

| Temperature (ºC) | Dispersed phage particles (%) | Aggregated phage particles (%) |
| --- | --- | --- |
| 25 | 90.91 ± 2.92 | 9.09 ± 0.94 |
| 30 | 92.55 ± 2.77 | 7.45 ± 1.32 |
| 35 | 94.54 ± 2.37 | 5.46 ± 1.19 |
| 40 | 91.33 ± 2.86 | 8.67 ± 1.41 |

**Supplementary Figure S3.** Exemplary AFM images of the C22 phage particles at 25 (a), 30 (b), 35 (c), and 40(d) ºC. The color gradient scale bar represented the height (Z-direction scale). The white scale bar was 100 nm.


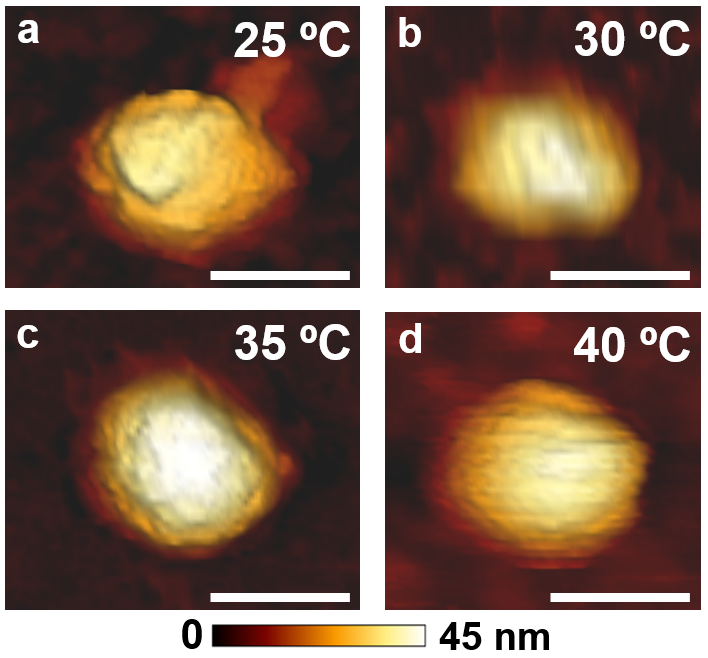


**Supplementary Table S3.** The average diameter of the C22 phage particles at 25, 30, 35, and 40 ºC. The error represented standard deviations (s.d.) of 50 particles.

| Temperature (ºC) | Average diameter (nm) |
| --- | --- |
| 25 | 39.9 ± 0.9 |
| 30 | 40.9 ± 1.3 |
| 35 | 40.7 ± 1.5 |
| 40 | 40.7 ± 0.5 |

**Supplementary Figure S4.** C22 phage particle (orange) was bound to an APTS-coated mica surface (grey). An AFM microcantilever moved downward to approach the particle and indented into the particle. The indentation on the particle and a mica surface yielded force-distance curves on the C22 phage particle and the mica surface for the stiffness calculation of the C22 phage.


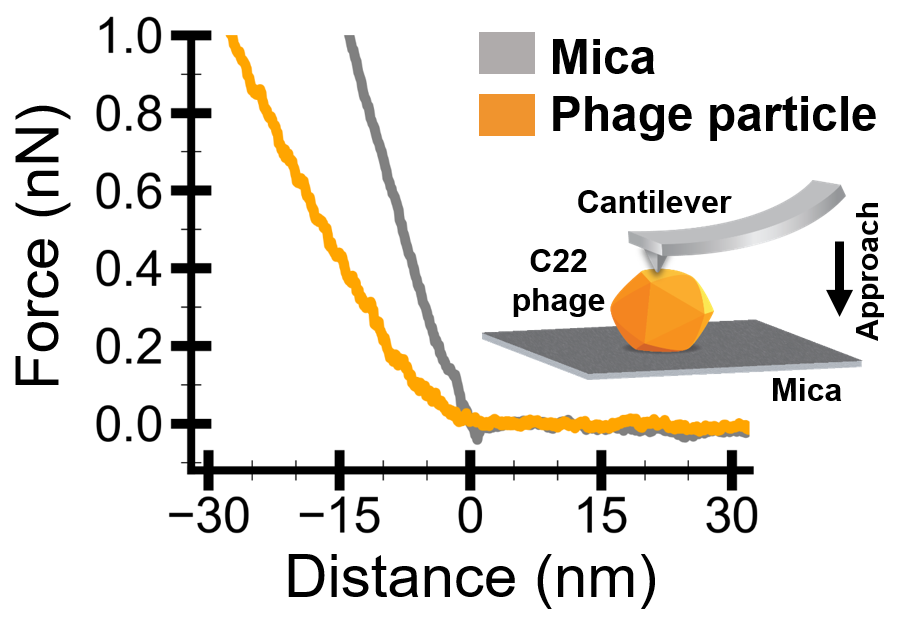

Supplement: Supplementary file 1 — Supplementary Information. [file 41598_2022_16795_MOESM1_ESM.docx]
